# Perceived Employability and Entrepreneurial Intentions Across University Students and Job Seekers in Togo: The Effect of Career Adaptability and Self-Efficacy

**DOI:** 10.3389/fpsyg.2019.00180

**Published:** 2019-02-08

**Authors:** Kokou A. Atitsogbe, Nambè P. Mama, Laurent Sovet, Paboussoum Pari, Jérôme Rossier

**Affiliations:** ^1^CePCO, Institute of Psychology, University of Lausanne, Lausanne, Switzerland; ^2^National Institute of Education Sciences, University of Lomé, Lomé, Togo; ^3^LATI, University Paris Descartes, Paris, France; ^4^NCCR LIVES, University of Lausanne, Lausanne, Switzerland

**Keywords:** career adaptability, general self-efficacy, self-perceived employability, entrepreneurial intentions, West Africa, Togo

## Abstract

This study examined the relationship between two personal resources, career adaptability and general self-efficacy, and two career outcomes, self-perceived employability and entrepreneurial intentions in a West African context, characterized by a developing economy. A Togolese sample of 334 university students and 216 job seekers completed French versions of the General Self-Efficacy Scale, the Self-Perceived Employability Scale, the Entrepreneurial Intentions Scale and an adapted form of the Career Adapt-Abilities Scale. A multi-group path analysis showed that the results are similar for both groups. Career adaptability and general self-efficacy were positively related to self-perceived employability. The contribution of career adaptability was especially strong for job seekers. Only general self-efficacy was related to entrepreneurial intentions. Furthermore, perceived employability was positively related in some way to entrepreneurial intentions in both groups. Career adaptability seems to be especially important for employability among job seekers (activation of resources), whereas entrepreneurial intentions may be more context-dependent. Finally, perceived employability failed to mediate the relationship between personal resources and entrepreneurial intentions in both samples.

## Introduction

The transition from education to work is considered a serious challenge for many young people, especially those with low social capital (Blustein et al., 2002). This transition is likely to be more difficult for university students and recent graduates facing extreme unemployment and other macro-level contextual barriers (Atitsogbe et al., 2016). Several theories and empirical investigations have stressed the important role of macro factors such as the economic context or public policies and micro factors such as personal resources that are thought to sustain individuals’ ability to interact with their environment, take control of situations, and produce significant changes in their careers and lives (Savickas et al., 2009; Duffy et al., 2016). Although both factors have been documented to have significant impacts on individuals’ career development, the effects of micro-level factors on career outcomes seem to be under-investigated in environments subject to uncertainty and economic constraints like Sub-Saharan Africa (Blustein et al., 2017). Given the evidence that future employability is a central concern for university students as well as recent graduates seeking work, and considering the fact that entrepreneurship could be an alternative in a context subject to extreme unemployment, it follows that personal resources could significantly contribute to vocational outcomes in such contexts. Consequently, this study investigated the usefulness of the personal resources of career adaptability and self-efficacy to perceived employability and entrepreneurial intentions in Togo, a sub-Saharan African country. Furthermore, it examined the relationship between perceived employability and entrepreneurial intentions, which has not been investigated to date in the vocational psychology literature.

## The Togolese Context

Togo is a French-speaking Sub-Saharan African country with a population of approximately 7 million inhabitants (United Nations, 2017). As OECD (2016) reported, the labor market in this country appears to be especially precarious, particularly for the youth. The unemployment rate is estimated at 32% for the global population; 28% of women and 21% of men who completed tertiary education were underemployed. According to the same report, one quarter of Togolese are facing difficulties in making the transition from education to satisfactory employment. For example, it takes average 35 months (without direct transition) for higher education graduates to access a first job; 40% of them relying on relationships or family to the detriment of classic job-searching strategies (OECD, 2016). Moreover, the country is classified a low-income country where informal employment stands at 63% while exceeding 70% in Sub-Saharan Africa (World Bank, 2019). From 2000, the number of students in higher education has tripled in 10 years and reached a total of 51,908 in 2011 for the two State Universities. This rapid growth in the number of students have significantly impacted the study conditions, leading to low success rates (42–56% with respect to the different fields and levels of study) (Chitou, 2011; OECD, 2016). Although Togo provides lower university tuition fees, only 12.3% of secondary school attendees will access higher education (OECD, 2016). In fact, Togolese University students face several contextual barriers such as a lack of financial means, unfavorable study conditions or perceived difficulties to integrate the labor market (Atitsogbe et al., 2016). Several researchers have suggested that higher education in this country should be reformed in order to facilitate young graduates’ occupational integration (e.g., Chitou, 2011).

In fact, occupational integration has become a major issue for both the government and the populations of this country. As in most Sub-Saharan African countries, the economic context has deteriorated since the early 1990s with the implementation of structural adjustment measures (e.g., budget reduction) recommended by the Bretton Woods Institutions (Gogué, 1997). Furthermore, in 1993, most of Togo’s international and bilateral funding partners suspended development cooperation for political reasons, aggravating the already precarious financial situation (Breuer and Asiedu, 2017). According to the Ministry of Planning and National Development, the informal sector represents 92.4% of the labor force while contributing 41.6% of the GDP in 2011. The informal sector has dramatic implications in terms of underemployment and working poverty. Moreover, the gap between the country’s development needs or labor market’s demands and the curriculum of the education system has contributed to the deregulation of the job market in a way that does not favor young higher-education graduates (Pieume, 2016).

The resumption of development cooperation with the European Union in the late 2000s, however, enabled the Government of Togo to start implementing measures in support of employment and entrepreneurship. Over the last few years, an entrepreneurship movement emerged in Togo through diverse national and international initiatives such as the *Foire des Jeunes Entrepreneurs* [Young Entrepreneurs Fair], which held its seventh meeting in August–September 2018 under the heading “quality and innovation.” Nevertheless, according to a joint report published by The African Development Bank Group [AfDB], The Organization for Economic Co-operation and Development [OECD], and The United Nations Development Programme [UNDP] (2017), this movement has not led to entrepreneurship enthusiasm yet, as the government has failed to implement sustainable and effective entrepreneurship education programs or policies that secure investments. For instance, Pari (2014) conducted a study among 360 job seekers registered at the National Employment Agency, finding that 85% of respondents reported preferring a paid job rather than creating their own business. Moreover, among job seekers who attended an entrepreneurship training program with the idea of creating their own business, 79% reported that they would prefer paid employment, although 56% showed high scores in motivation to be an entrepreneur. These findings led the author to conclude that the reluctance of job seekers for entrepreneurial careers is due to contextual factors that may fail to facilitate business creation. In line with Pari (2014) findings, Golo (2012) reported factors such as a lack of financial means, difficulties to access credit, unfavorable taxation, and increased competition as inhibitors of entrepreneurial intentions in potential entrepreneurs. However, according to the OECD (2016), in contexts that are characterized by a turbulent job market, difficulties in job accessibility, uncertainty and a lack of sustainable employment as Togo, entrepreneurship could be an alternative for the unemployed and contribute to the development of new sectors of the economy. Therefore, investigating entrepreneurial intentions and perceived employability among university students and recent graduates might provide new insights into the occupational integration challenges of these populations.

## Perceived Employability and Entrepreneurial Intentions

### Self-Perceived Employability

Rothwell et al. (2008, p. 2) defined self-perceived employability as “the perceived ability to obtain sustainable employment appropriate to one’s qualification level.” Among the broad range of variables that influence self-perceived employability, personal resources appear to play a significant role. In this respect, Kasler et al. (2017) reported a significant and positive association between hope, grit and self-perceived employability. Ngo et al. (2017) stressed a significant predictive role of general self-efficacy and de Guzman and Choi (2013), the role of career adaptability. Moreover, a longitudinal study conducted by Berntson et al. (2008) showed that self-perceived employability and self-efficacy were associated with each another and that self-perceived employability preceded self-efficacy. The findings of this latter study should be considered in light of an employed population, as the sample considered for the study essentially consisted of employed individuals with enriched working experience. Regarding university students and newly graduated employment-seekers, it would be reasonable to expect that self-perceived employability would be preceded by general self-efficacy, considering their limited work experience. Perceived employability has been linked to contextual antecedents such as job insecurity (e.g., Mäkikangas et al., 2013) and to interpersonal variables such as support (Forsythe, 2017). Several empirical studies have shown the significant contribution of perceived employability to other variables such global health including psychological functioning, job search behaviors, or job satisfaction (e.g., Berntson and Marklund, 2007; Gowan, 2012; Onyishi et al., 2015). It has also been found to serve as a strong moderator/mediator between labor market state variables and well-being, and between self-evaluations and job search behaviors (e.g., Silla et al., 2009; Onyishi et al., 2015).

Multiple conceptualizations of employability reflecting the interplay between individual characteristics and contextual factors have been documented (for a review, see Guilbert et al., 2016). Some of these models placed significant weight on one or the other of these two factors. For example, Yorke (2006) argued that graduates’ employability largely depends on the attended higher education institution in that, the more training they were provided meet the labor market requirements, the more likely they are to gain employment and achieve career success. Moreover, Berntson and Marklund (2007) focused on individual characteristics such as their skills, experience, network, personality and knowledge of the labor market. One of the individual-centered and frequently used model of employability is the one developed by Rothwell et al. (2008). A part from the self-belief dimension which assess the perceived ability to gain employment, this model includes three external dimensions related to the prestige of the university attended, the field of study, and the state of the external labor market. The main advantage of this model is that it covers both internal and external dimensions of employability. Although most studies that used this model have been conducted among the student population, we found it to be usable across young graduates seeking employment given that its four dimensions could significantly account for employability across this population.

### Entrepreneurial Intentions

There has been a substantial interest in entrepreneurship in recent years, especially in low-income countries, where it has been found to be an important factor for economic growth, productivity, and social development (Denanyoh et al., 2015; OECD, 2016; Campos et al., 2017). In the same manner, there is a growing interest in investigating entrepreneurial intentions among university students in such contexts. Entrepreneurial intentions are defined as the intention of individuals to start their own business (Yıldırım et al., 2016). More recently, by assessing the determinants of entrepreneurial intentions among Ghanaian polytechnic tertiary students, Denanyoh et al. (2015) reached the conclusion that educational, familial, and structural supports significantly contribute to entrepreneurial intentions. Although job seekers showed a motivation to become entrepreneurs in Togo, they usually prefer a paid job; their entrepreneurial motivation seems to be weakened by limiting contextual factors such as difficulties to access loans, a lack of favorable policies in favor of startups among others (Pari, 2014). It has been demonstrated that personal resources such as general self-efficacy and career adaptability have a positive influence on entrepreneurial intentions (Boyd and Vozikis, 1994; Tolentino et al., 2014). However, almost no studies have investigated such relationships in developing or emerging economies. A concept similar to self-efficacy that has been linked to entrepreneurial intentions in the majority of studies is entrepreneurial perceived behavioral control. In his theory of planned behavior, Ajzen (1991, p. 183) referred to perceived behavioral control as “people’s perception of the ease of performing the behavior of interest.”. Hence, entrepreneurial perceived behavioral control is viewed as the perceived feasibility of performing entrepreneurial behaviors. Indeed, the concept of perceived behavioral control was found to overlap with Bandura’s conceptualization of self-efficacy (Krueger et al., 2000), which has been used in several studies (Belchior and Liñán, 2017).

### The Link Between Self-Perceived Employability and Entrepreneurial Intentions

Both self-perceived employability and entrepreneurial intentions have been linked separately to several variables in the career literature (Liñán and Fayolle, 2015; Kasler et al., 2017). Furthermore, these two career outcomes have been linked to the similar antecedent such as self-efficacy (e.g., Boyd and Vozikis, 1994; Ngo et al., 2017). However, to our knowledge, no empirical studies have systematically examined the relationship between the two constructs. Moreover, there is no explicit theoretical framework that elucidates the possible links between these two career variables. Despite these limitations, we sought to formulate a hypothesis based on those variables regarding a context characterized by high unemployment and adverse economic situations. It is obvious that employable individuals are likely to implement career development behaviors as reviewed by Guilbert et al. (2016). Berntson et al. (2008, p. 414) stated that high levels of perceived employability “reflect one’s ability to solve specific work-related problems and handle difficult situations.” Moreover, psychology of working theorists have stressed that individuals in difficult socio-economic conditions could reach successful employment if they activate resources such as career adapt-abilities (Duffy et al., 2016). For these reasons, we argue that extreme economic conditions characterized by high levels of unemployment could lead people with an especially high self-perceived employability to create their own employment to integrate into the labor market, and for this reason be characterized by higher entrepreneurial intentions. Therefore, we posit that in developing economies such as Togo, higher levels of self-perceived employability may foster individuals’ intentions to implement entrepreneurial behaviors.

## Personal Resources

### The Role of Career Adaptability

The career construction theory (CCT; Savickas, 2005) stresses the importance of past and present environments and how individuals interact with these environments to produce significant changes in their lives. As a key concept of the theory, career adaptability is considered as a set of psychosocial processes that help individuals manage their own careers—for example, to prepare for or master school-to-work transitions—and design their lives. Career adapt-abilities include career concern, control, curiosity, and confidence (Savickas and Porfeli, 2012). *Career concern* denotes the extent to which individuals are interested in their future career and are preparing for it. *Career control* reflects self-discipline and agency and, therefore, the ability of individuals to self-manage their career. *Career curiosity* refers to individuals’ willingness to learn more about their desired domains or occupations, with the idea of searching for a better person-job fit. *Career confidence* denotes the degree to which they believe in their ability to successfully address barriers they encounter while achieving their career goals. Rossier (2015a) argued that adapt-abilities are an important set of resources among others, such as emotion regulation skills or self-efficacy that have been studied by approaches such as the social cognitive career theory (SCCT). There is a widespread agreement in the literature that career adaptability is significantly associated with several person-, career-, and work-related variables that can be grouped into four categories including adaptivity variables, adapting response variables, adaptation result variables, and socio-demographical characteristics (Rudolph et al., 2017). In this study, we supposed that these resources, if mobilized, should help university students develop a clear vision of their future and facilitate their transition to the world of work. Accordingly, such resources should increase the employability of job-seekers and help them find or even create their own opportunities in job-insecure and economically constrained environments.

Savickas and Porfeli (2012) have developed a measure to assess the four career adaptability resources, the Career Adapt-Abilities Scale (CAAS), which has demonstrated a strong validity across different cultures. Several cross-cultural researchers suggested that measures developed in one context should be adapted for use in other contexts considering local specifies (e.g., Van de Vijver, 2016). In line with this recommendation, the CAAS will be slightly adapted to the Togolese context in the present study.

### The Role of General Self-Efficacy

Bandura (1977, p. 193) referred to self-efficacy expectations as “a mechanism of operation” that involves one’s conviction to successfully implement actions that will lead to the desired outcome. According to the SCCT, self-efficacy is one of the two mediators that account for career development outcomes, namely, interest development, choice making, and performance attainment (Lent et al., 1994). A recent extension of the SCCT, the Social Cognitive Model of Career Self-Management stressed the contribution of personal resources such as self-efficacy to several career outcomes (Lent and Brown, 2013). Based on this perspective, self-efficacy has been intensively studied over the two past decades (e.g., Lent et al., 2017). General self-efficacy is referred to as “the belief in one’s competence to cope with a broad range of stressful or challenging demands” (Luszczynska et al., 2005, p. 439). It appears to impact intention, intention implementation, outcome expectancies, and self-regulation as reviewed by Luszczynska et al. (2005). It is positively correlated with career adaptability (Öncel, 2014). Available studies demonstrate that higher levels of general self-efficacy are associated with higher levels of perceived employability (e.g., Ngo et al., 2017). Moreover, a study conducted among Taiwanese agricultural college students showed that higher levels of general self-efficacy were associated with higher levels of entrepreneurial intentions (Wang et al., 2016). Some researchers stressed the fact that the self-efficacy construct should be domain-specific (Betz and Hackett, 2006). Accordingly, the relationship between self-efficacy tied to specific domains or tasks and career-related variables has come under investigation. For example, entrepreneurial self-efficacy has been linked to entrepreneurial intention in most studies (e.g., Wilson et al., 2007). However, in this study the aim was to assess the impact of self-efficacy on both employability and entrepreneurial intentions. For this reason, general self-efficacy will be considered. It has been well documented that the development of employability on one hand, and entrepreneurship on the other hand, are two challenging and complex problem-solving situations (Wilson et al., 2007; Guilbert et al., 2016). Both depend on individual factors such as formal and actual competence, interpersonal skills or personal characteristics, and contextual factors such as the political, social, and economic situations (Nilsson, 2010; Guilbert et al., 2016). Therefore, the achievement of these two career outcomes and even intentionality regarding them would involve abilities or perceived abilities to cope with various related external challenges or tasks. Thus, we found general self-efficacy to better reflect such perceived broad abilities that could account for both career outcomes simultaneously.

## The Aims of the Study

This study aimed at investigating the effect of career adaptability and self-efficacy on two vocational outcomes, self-perceived employability and entrepreneurial intentions across university students and job seekers in a context subject to extreme unemployment and economic constraints. Furthermore, the study investigated for the first time the link between self-perceived employability and entrepreneurial intentions, and explored if perceived employability would mediate between resources and entrepreneurial intentions. University students and recent graduates seeking work are two populations that are not at the same level regarding the transition from education to work. Given the fact that job seekers are facing occupational integration challenges and certain social pressures, it is likely that adapt-abilities have been significantly activated in this sub-population as observed by Rossier (2015b). Moreover, it has been well documented that perceived employability significantly mediates the relationship between core self-evaluations, which are beliefs about one’s capabilities, and career outcomes such as job search behaviors (e.g., Onyishi et al., 2015). While personal resources are expected to be associated with entrepreneurial intentions, it is likely that perceived employability will mediate in this relationship. Based on the above literature and rationale, we hypothesized that career adaptability and general self-efficacy would have direct significant and positive paths on both perceived employability and entrepreneurial intentions in both groups (H1). However, we expect career adaptability to exhibit a stronger effect in job seekers (H2). We also hypothesized that self-perceived employability will be positively related to entrepreneurial intentions in both groups (H3) and will mediate between resources and entrepreneurial intentions (H4).

## Methods

### Participants

A sample of 557 adults were surveyed. Seven profiles including two university students and five job seekers with more than 10% of missing data were excluded. The remaining 550 profiles (67.1% men and 32.9% women), aged 18 to 44 (*M* = 25.11, *SD* = 4.42) was considered for the study. Among this sample, 334 (60.7%) were university students (87.7% bachelor level and 12.3% master’s) enrolled in different majors and 216 (39.3%) were job seekers (91.2% with a bachelor degree and 8.8% a master’s degree) who had graduated from tertiary education and were registered at the National Employment Agency. More precisely, 77 (23.1%) students against 70 (32.4%) job seekers enrolled or graduated in economy, business or technical fields. Moreover, 257 (76.9%) students against 123 (56.9%) job seekers enrolled or graduated in social sciences or other fields, and 23 (10.6%) of job seekers did not provide such information. The job seeking duration for young graduates ranged from 1 to 168 months (*M* = 25.28, *SD* = 27.83) with a median of 12 months.

### Instruments

#### Career Adapt-Abilities

Career adapt-abilities were assessed using the French version of the Career Adapt-Abilities Scale (CAAS; Savickas and Porfeli, 2012; Johnston et al., 2013). This scale includes 24 items divided into four subscales (concern, control, curiosity, and confidence), with six items each. To adapt the CAAS to the Togolese context, one item— “develop relations and networks”—was added to the confidence subscale. This was because being able to benefit from a social network was believed to be especially relevant to the access of employment in Togo which is a difficult context regarding employment as reported by OECD (2016). The Togolese-version of the CAAS thus consisted of 25 items indicating abilities, which participants were asked to rate on a 5-point Likert-type scale from 1 (I don’t have the ability/This is not a resource for me) to 5 (I have a very strong ability/This is a very important resource for me). Responses are computed in terms of the four subscales as well as a global score. Internal reliabilities for this Togolese version for the total sample, the university students, and the job seekers were, respectively, 0.75, 0.73, and 0.77 for concern, 0.72, 0.70, and 0.73 for control, 0.78, 0.72, and 0.84 for curiosity, 0.83, 0.80, and 0.84 for confidence, and 0.91, 0.89, and 0.92 for the total career adaptability score.

#### Self-Efficacy

General self-efficacy in terms of personal beliefs was assessed using the French version of the General Self-Efficacy Scale (Schwarzer and Jerusalem, 1995; Scholz et al., 2002). The scale is currently available in 33 languages and consists of 10 items measured on a 5-point Likert scale with scores ranging from 1 (not at all true) to 4 (very true). This scale is the most commonly used to assess general self-efficacy around the world and has shown strong measurement stability across cultures (Luszczynska et al., 2005). Scholz et al. (2002) reported internal reliability coefficients ranging from 0.75 to 0.91 across 25 countries. Internal reliability coefficients for the total sample, university students, and job seekers were 76, 0.77, and 0.76, respectively, close to values reported by Scholz and colleagues for some countries (e.g., India, α = 0.75 or Portugal, α = 0.76).

#### Employability

Self-perceived employability was measured using the student version of the Self-Perceived Employability Scale (Rothwell et al., 2008), which was translated into French using a back-translation method with permission from the authors (see Appendix 1 for the French version of this scale). The scale, consisting of 16 items that evaluate four basic components related to employability and their interactions, can be used with students and job-seekers. The four basic components address the prestige of the university attended, the field of study, the state of the external labor market, and beliefs about one’s ability to obtain a job. Rothwell et al. (2008) reported an internal reliability coefficient of 0.75 for a university student sample in the United Kingdom. In Togo, we found internal reliability coefficients of 0.76, 0.75, and 0.78 for the overall sample, university students, and the job seekers, respectively.

#### Entrepreneurial Intentions

The French-translation (A. Battistelli, personal communication, November 21, 2015) of the Entrepreneurial Intention Scale (Liñán and Chen, 2009) was used for this study. This scale consists of six pure-intention items that evaluate a participant’s determination to implement entrepreneurial behaviors and to become an entrepreneur. A 5-point Likert scale with scores ranging from 1 (completely disagree) to 5 (completely agree) have been used (Battistelli, 2001) instead of the 7-point Likert scale used in an early study by Liñán and Chen (2009). The reliabilities of this scale for the total sample, the university student subsample, and the job seeker subsample were all α = 0.87.

### Procedure

University students were recruited from several departments at the University of Lomé. In each department, response sessions were organized under the supervision of the fourth author working at that university. Questionnaires were paper-pencil. Job seekers were recruited at the National Employment Agency in Lomé, within the framework of a training designed for new graduates looking for occupational integration. The second author was responsible for data collection from job seekers. Response sessions were organized in collaboration with the staff of the Agency. In the same manner as that of the university students, participants responded to a paper-pencil questionnaire. According to Togo national guidelines, questionnaire based research conducted by public institutions does not currently require ethics committee approval. However, we respected the codes of practice and ethics in research and in particular the Declaration of Helsinki. Accordingly, all participants provided informed written consent and volunteered for this study.

### Analyses

A total of 557 participants completed the paper-pencil questionnaire. Seven cases were deleted due to being over the cut off of 10% missing data (Bennett, 2001). For the remaining respondents, missing data were handled using a multiple imputation technique in the R package “MICE” (for more detail about this procedure, see Schlomer et al., 2010).

Descriptive statistics including the means (*M*), standard deviations (*SD*), internal reliabilities, inter-correlations, skewness (*S*), and kurtosis (*K*) were computed for all scales using SPSS 24.0 software. Scale normality was evaluated by means of *S* and *K*, whose values should be equal to or below an absolute value of 2. Scale internal consistency was assessed using Cronbach’s alpha.

Structural validity was assessed for each construct by means of confirmatory factor analyses (CFA) using AMOS statistical package 24.0, with maximum likelihood estimation. Various model fit indices were analyzed: χ^2^ per degree of freedom (χ^2^/*df*), the goodness of fit index (GFI), the comparative fit index (CFI), the Tucker–Lewis index (TLI), and the root mean square error of approximation (RMSEA). According to Bollen and Long (1993), values of χ^2^/df ≤ 3, GFI, CFI, and TLI ≥ 0.90, and RMSEA ≤ 0.05 indicate that a specified model has exhibited an adequate fit. Regarding the career adaptability construct, based on the etic–emic approach, one item, “develop relations and networks,” that was found to be relevant in the Togolese context was added to the CAAS to be adapted to this context (Van de Vijver, 2016). Item-to-dimension reliabilities were *r* = 0.29 for concern, 0.31 for control, 0.40 for curiosity and 0.43 for confidence. Hence, this item was added to the confidence subscale of the CAAS-Togo form.

Furthermore, convergent, discriminant and incremental validity were assessed for the CAAS-Togo. As Weiner and Greene (2007) stated, convergent validity evaluates how well a measure correlates with other measures measuring similar construct whereas discriminant validity evaluates how unrelated a measure is to other measures measuring dissimilar constructs. Moreover, for these authors, incremental validity should address whether the scale of interest provides additional information after having considered the predictive power of previous variables.

Finally, four-factor measurement models of the overall data and of the two groups were assessed, with career adaptability, general self-efficacy, self-perceived employability, and entrepreneurial intentions considered as latent variables. In this respect, the four career adapt-abilities (i.e., concern, control, curiosity, and confidence) were assessed for the observed variables. As item-level models usually result in large modification indices and poor fit — needing an increase of covariance links between related error terms to obtain substantial improvement of the fit—, item parceling solution was considered for some scales in defining our hypothesized model as recommended by Thompson and Melancon (1996). Items were parceled into the four basic components of self-perceived employability and empirically by means of factor analysis to reduce the observed variables to three for general self-efficacy and to four for entrepreneurial intentions. The reduction of observed variables increases the robustness of the model (e.g., Berntson et al., 2008; De Vos and Soens, 2008). To test the hypothesized paths for the university student and job seeker groups, a multi-group path analysis was conducted.

## Results

### Descriptive Statistics

Means, standard deviations, skewness, kurtosis, internal reliabilities, and inter-correlations for all scales and per group are presented in Table 1. All correlations between career adaptability, general self-efficacy, self-perceived employability, and entrepreneurial intentions were positive and significant for both students and job seekers. With respect to the career outcomes, both groups did not differ for perceived employability but job seekers scored higher on entrepreneurial intentions (*d* = 0.36). Considering entrepreneurial intentions, no differences were observed between students enrolled in economy, business or technical majors and those enrolled in social sciences or other majors. Similar results were found across job seekers. With respect to gender, men scored higher than women on entrepreneurial intentions only in the job seeker group (*d* = 0.31). Finally, there were no differences on entrepreneurial intentions between graduates that had been seeking job for a relatively long time and those who had been for a relatively short time (more or less than the median of 12 months).

**Table 1:** Descriptive statistics, internal reliabilities, and inter-correlations for career adapt-abilities, GSE, SPE, and EI for both sub-samples.

	Students (*n* = 334)		Job seekers (*n* = 216)						Correlations							
Scale	*M*	*SD*	*M*	*SD*	*d*	*S*	*K*	α	1	2	3	4	5	6	7	8
(1) CA	3.75	0.50	3.97	0.54	−0.43^∗∗∗^	−0.15	−0.14	0.91	−	0.76^∗∗∗^	0.85^∗∗∗^	0.87^∗∗∗^	0.86^∗∗∗^	0.38^∗∗∗^	0.38^∗∗∗^	0.18^∗∗^
(2) Concern	3.80	0.64	3.96	0.63	−0.25^∗∗^	−0.24	−0.40	0.75	0.76^∗∗∗^	−	0.56^∗∗∗^	0.53^∗∗∗^	0.50^∗∗∗^	0.27^∗∗∗^	0.26^∗∗∗^	0.20^∗∗^
(3) Control	3.91	0.60	4.08	0.61	−0.29^∗∗^	−0.54	0.15	0.72	0.80^∗∗∗^	0.51^∗∗∗^	−	0.66^∗∗∗^	0.65^∗∗∗^	0.30^∗∗∗^	0.29^∗∗∗^	0.09
(4) Curiosity	3.54	0.63	3.74	0.71	−0.31^∗∗∗^	−0.04	−0.27	0.78	0.81^∗∗∗^	0.47^∗∗∗^	0.53^∗∗∗^	−	0.68^∗∗∗^	0.33^∗∗∗^	0.38^∗∗∗^	0.17^∗^
(5) Confidence	3.75	0.62	4.08	0.63	−0.52^∗∗∗^	−0.32	−0.27	0.83	0.83^∗∗∗^	0.47^∗∗∗^	0.54^∗∗∗^	0.59^∗∗∗^	−	0.36^∗∗∗^	0.34^∗∗∗^	0.15^∗^
(6) GSE	3.25	0.46	3.29	0.44	−0.09	−0.56	0.27	0.76	0.46^∗∗∗^	0.37^∗∗∗^	0.35^∗∗∗^	0.30^∗∗∗^	0.43^∗∗∗^	−	0.28^∗∗∗^	0.20^∗∗^
(7) SPE	3.49	0.59	3.54	0.64	−0.09	−0.21	0.06	0.76	0.31^∗∗∗^	0.26^∗∗∗^	0.22^∗∗∗^	0.24^∗∗∗^	0.27^∗∗∗^	0.28^∗∗∗^	−	0.19^∗∗^
(8) EI	3.89	0.87	4.20	0.86	−0.36^∗∗∗^	−0.89	0.29	0.87	0.14^∗^	0.13^∗^	0.06	0.08	0.17^∗∗^	0.20^∗∗∗^	0.14^∗^	−

*CA, Career adaptability; GSE, General self-efficacy; SPE, Self-perceived employability; EI, Entrepreneurial intentions. Cohen’s ds are negative when job seekers scored higher than university students. S, skewness; K, kurtosis; α, Cronbach’s alpha coefficient. Correlations for students are reported above the diagonal. Correlations for job seekers are presented below the diagonal. ^∗^p < 0.05, ^∗∗^p < 0.01, ^∗∗∗^p < 0.001.*

### Structural Validity of Instruments and Measurement Model

Based on the theoretical model of the CAAS (Savickas and Porfeli, 2012), a four-factor structure was tested for both the French language form (Johnston et al., 2013) and the Togo form of the CAAS considering the overall sample. Four first-order latent variables (i.e., concern, control, curiosity, and confidence) and a second-order construct (career adaptability) were considered. Fit indices for the initial model tested for the CAAS French language form indicated a modest fit: χ^2^(248) = 752.53, *p* < 0.001, χ^2^/*df* = 3.03, GFI = 0.894, CFI = 0.877, TLI = 0.863, and RMSEA = 0.061. Those for the CAAS Togo-form including the added item were slightly better: χ^2^(271) = 781.15, *p* < 0.001, χ^2^/*df* = 2.88, CFI = 0.894, CFI = 0.879, TLI = 0.866, and RMSEA = 0.059. Both models were adjusted by setting four covariances between error terms that had modification indices (MI) equal or above 20 in each case (Johnston et al., 2013). As reported in Table 2, adjusted models’ fit indices were adequate or close to the expected values for the CAAS French language form, χ^2^(244) = 536.65, *p* < 0.001, χ^2^/*df* = 2.20, GFI = 0.922, CFI = 0.928, TLI = 0.919, and RMSEA = 0.047 and the CAAS-Togo, χ^2^(267) = 567.01, *p* < 0.001, χ^2^/*df* = 2.12, GFI = 0.922, CFI = 0.929, TLI = 0.920, and RMSEA = 0.045. As can be seen, the CAAS-Togo exhibited a better fit to the overall data compared to the CAAS French language form. We concluded that the added item increased the robustness of the scale. The standardized loadings of the CAAS-Togo ranged from 0.43 to 0.72 for the items (*Mdn* = 0.61) and from 0.76 to 0.93 (*Mdn* = 0.86) for the second order variables. These loadings were comparable to those of the French-language form of the CAAS with item loadings ranging from 0.56 to 0.73 (*Mdn* = 0.61) and from 0.77 to 0.88 (*Mdn* = 0.81) for the second-order variables as reported by Johnston et al. (2013).

**Table 2:** Structural validity of the measuring instruments and fit indices of the hypothesized model.

Adjusted model	χ^2^	*df*	χ^2^/*df*	*p*	GFI	CFI	TLI	RMSEA
CAAS French language form (4-factor model, MI > 20)	536.65	244	2.20	<0.001	0.922	0.928	0.919	0.047
CAAS Togo-form (4-factor model, MI > 20)	567.01	267	2.12	<0.001	0.922	0.929	0.920	0.045
Generalized self-efficacy (unidimensional, MI > 10)	89.10	32	2.78	<0.001	0.968	0.938	0.913	0.057
Self-perceived employability (4-factor model, MI > 10)	170.55	94	1.81	<0.001	0.962	0.940	0.923	0.039
Entrepreneurial intentions (unidimensional, MI > 20)	14.55	6	2.42	<0.001	0.992	0.994	0.986	0.051
Hypothesized 4-factor model	152.11	84	1.81	<0.001	0.965	0.973	0.966	0.038
Hypothesized 4-factor model (multi group)	241.32	170	1.42	<0.001	0.947	0.971	0.965	0.028

*N = 550. CAAS, career adapt-abilities.*

Furthermore, structural validity was also tested for the other instruments. Unidimensional measurement was assessed for the GSE scale (Scholz et al., 2002). Fit indices for the initial model were: χ^2^(35) = 166.73, *p* < 0.001, χ^2^/*df* = 4.76, GFI = 0.941, CFI = 0.858, TLI = 0.817, and RMSEA = 0.083. This model was improved by setting three covariation links between error terms associated with MI equal or above 10, leading to acceptable fit indices: χ^2^(32) = 89.10, *p* < 0.001, χ^2^/*df* = 2.78, GFI = 0.968, CFI = 0.938, TLI = 0.913, and RMSEA = 0.057. A four-factor structure was tested for the Self-Perceived Employability Scale (Rothwell et al., 2008). The fit of the initial model to data was: χ^2^(98) = 261.26, *p* < 0.001, χ^2^/*df* = 2.66, GFI = 0.942, CFI = 0.871, TLI = 0.843, and RMSEA = 0.055. The model was improved considering the MI equal or above 10 criteria. This allowed setting four covariance links in the model, which subsequently improved the fit: χ^2^(94) = 170.55, *p* < 0.001, χ^2^/*df* = 1.81, GFI = 0.962, CFI = 0.940, TLI = 0.923, and RMSEA = 0.039. The Entrepreneurial Intention Scale showed mitigated fit indices regarding the initial unidimensional model tested: χ^2^(9) = 112.89, *p* < 0.001, χ^2^/*df* = 12.54, GFI = 0.940, CFI = 0.931, TLI = 0.885, and RMSEA = 0.145. Adjustments applying the MI equal or above 20 criteria led to setting three covariance links in the model, which led to good fit: χ^2^(6) = 14.55, *p* < 0.001, χ^2^/*df* = 2.42, GFI = 0.992, CFI = 0.994, TLI = 0.986, and RMSEA = 0.051.

Finally, a measurement model that simultaneously considered career adaptability, general self-efficacy, self-perceived employability, and entrepreneurial intentions was first tested for the overall sample with four indicators for career adaptability, self-perceived employability, and entrepreneurial intentions, and three indicators for general self-efficacy. The model fit the overall data well: χ^2^(84) = 152.11, *p* < 0.001; χ^2^/*df* = 1.81; GFI = 0.965; CFI = 0.973; TLI = 0.966; and RMSEA = 0.038. Likewise, a multi-group CFA showed that the measurement model exhibited adequate fit indices for the two subgroups: χ^2^(170) = 241.32, *p* < 0.001; χ^2^/*df* = 1.42; GFI = 0.947; CFI = 0.971; TLI = 0.965; and RMSEA = 0.028. Fit indices for all instrument and the measurement model considering the overall sample are summarized in Table 2.

### Convergent, Discriminant and Incremental Validity of the CAAS-Togo

**FIGURE 1:**
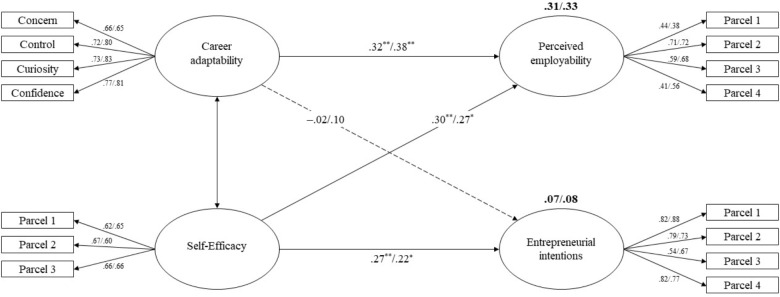
Hypothesized model with standardized estimates for the university students and the job seekers, respectively. Non-significant paths are shown in a broken line. In bold are the amount of variance explained for perceived employability and entrepreneurial intentions across both groups. ^∗^*p* < 0.05, ^∗∗^*p* < 0.01.

As shown in Table 1, correlations between overall career adaptability and the measure of general self-efficacy were 0.38 and 0.46 (both *p* < 0.001) for the student and job seeker groups, respectively, suggesting a relatively high convergence between the two constructs. Career adaptability and entrepreneurial intentions are correlated to a lesser extent across both groups, respectively (*r* = 0.18, *p* < 0.01; *r* = 0.14 *p* < 0.05). Moreover, as shown in Figure 1, both constructs were found to be unrelated, suggesting discriminant validity of career adaptability. Finally, we performed a hierarchical regression in order to assess the incremental validity of career adaptability over general self-efficacy in predicting perceived employability. Age and gender were entered in a first step, followed by general self-efficacy in a second step to evaluate their contribution to perceived employability. In a third step, previous steps were continued adding the overall career adaptability score to evaluate the incremental validity of career adaptability over general self-efficacy. After controlling for age and gender, self-efficacy explained 7 and 8% of perceived employability variance for university students and job seekers, respectively. The additional effect of career adaptability was particularly important in job seekers, Δ*R*^2^ = 0.09, *F*(2,213) = 21.47, *p* < 0.001 compared to university students, Δ*R*^2^ = 0.04, *F*(2,331) = 22.49, *p* < 0.001. These findings suggested that career adaptability had a significant incremental validity regarding the predictive power of self-efficacy on perceived employability.

### From Personal Resources to Career Outcomes

A multi-group path analysis yielded fit indices identical to those yielded for the measurement model: χ^2^(170) = 241.32, *p* < 0.001; χ^2^/*df* = 1.42; GFI = 0.947; CFI = 0.971; TLI = 0.965; and RMSEA = 0.028. Career adaptability and self-efficacy explained 31 and 32% of the variance in perceived employability, and 7 and 8% of the variance in entrepreneurial intentions for the student and the job seeker groups, respectively. As expected and with respect to the two groups, career adaptability and general self-efficacy produced significant direct and positive paths to self-perceived employability across the university student group (β = 0.32, *p* < 0.01; β = 0.30, *p* = 0.01, respectively) and the job seeker group (β = 0.38, *p* < 0.01; β = 0.27, *p* = 0.05, respectively) as reported in Figure 1. This suggests that career adaptability and general self-efficacy are significant predictors of self-perceived employability, confirming hypothesis 1 with respect to self-perceived employability. Furthermore, general self-efficacy produced significant direct and positive paths to entrepreneurial intentions across university students (β = 0.27, *p* = 0.01) and the job seekers (β = 0.22, *p* < 0.05), indicating that general self-efficacy significantly predicted entrepreneurial intentions. However, the paths from career adaptability to entrepreneurial intentions was non-significant for either the student (β = -0.02, *p* = 0.86) or the job seeker group (β = 0.10, *p* = 0.32), indicating that entrepreneurial intentions are not predicted by career adaptability. Self-efficacy served as the unique significant predictor of entrepreneurial intentions in the two groups suggesting that hypothesis 1 was partially confirmed with respect to entrepreneurial intentions.

Regarding hypothesis 2, as expected, career adaptability exhibited a stronger effect than self-efficacy (β = 0.38 vs. β = 0.22) in predicting perceived employability across the job seeker group. However, the assumption regarding the detrimental effect of career adaptability on self-efficacy in predicting entrepreneurial intentions was not supported, suggesting a partial confirmation of hypothesis 2.

### Self-Perceived Employability and Entrepreneurial Intentions

Finally, correlations between perceived employability and entrepreneurial intentions were positive across the university student group (*r* = 0.19, *p* < 0.01) and the job seeker group (*r* = 0.14, *p* < 0.05). Moreover, self-perceived employability was found to covary with entrepreneurial intentions in both groups, respectively, after controlling for sex in the job seeker group (β = 0.16, *p* = 0.032; β = 0.22, *p* = 0.021). Age and the level of education attained were not controlled as they were not related to the outcome variable in any group. Results suggest that perceived employability was positively related to entrepreneurial intentions across both groups as expected, confirming hypothesis 3. Furthermore, and to test hypothesis 4, we evaluated if an additional path from perceived employability to entrepreneurial intentions would be significant using 5,000 bootstrap samples with 95% bias-corrected confidence intervals (Shrout and Bolger, 2002). The comparison of this model to the previous one (i.e., Figure 1) revealed that the additional path did not significantly improve the model [Δχ^2^(2) = 1.82, *p* = 0.40]. Considering that this additional path and the paths between resources and entrepreneurial intentions were non-significant across the two groups suggest that perceived employability cannot be considered as a mediator.

## Discussion

This is the first study that investigated the impact of personal resources of career adaptability and self-efficacy on two career outcomes (i.e., self-perceived employability and entrepreneurial intentions) in the Togolese context, with the particular aspect of comparing findings for university students and job seekers, two populations that are at different levels regarding the transition from university to work. Furthermore, the link between self-perceived employability and entrepreneurial intentions, which has not been investigated to date was evaluated for the two subsamples. The CAAS-Togo form and the other instruments fit the data well. The measurement model exhibited good fit indices for the overall data as well as for the two subsamples. Likewise, path analyses yielded good fit indices for the overall data and the subsamples. Thus, hypothesized direct paths were tested.

### Psychometric Properties of the Measuring Instruments

This study provided an adaptation of the Career Adapt-Abilities Scale to the Togolese context. Our findings clearly indicated that this scale exhibited good fit indices and showed adequate convergent, discriminant, and incremental validity. Therefore, we conclude that the CAAS-Togo is a valid and useful measure to assess career adaptability in a Sub-Saharan Africa context. The other scales such as the General Self-Efficacy Scale, the Self-Perceived Employability Scale, and the Entrepreneurial Intentions Scale fit the Togo data well and have shown a strong structural validity across our overall sample. This suggests that these instruments can be used in this context and will contribute filling the gap of the lack of measures in most of Sub-Saharan Africa countries, which is one major limitation for researchers in this area (Atitsogbe et al., 2016).

### Effects of Personal Resources on Career Outcomes

Consistent with our expectations, significant and positive direct paths were observed from career adaptability and general self-efficacy to self-perceived employability, for both subsamples. This suggests that the extent to which students and recent graduates seeking employment are confident in their readiness to cope with the university-to-work transition (career adaptability) and/or with a broad range of stressful or challenging demands (general self-efficacy) directly relates to their perceived ability to find a job (self-perceived employability). Our findings contradict those of Coetzee and Oosthuizen (2012), where the self-efficacy of adult workers enrolled in undergraduate studies did not relate significantly to their perceived employability. However, the findings of our investigation support the important role of personal resources, as highlighted by social cognitive career theorists and career construction theorists, and reaffirm the conclusions of several empirical studies regarding the positive influence of the above-mentioned personal resources on the self-perception of employability (e.g., Lent et al., 1994; Savickas, 2005; de Guzman and Choi, 2013). Moreover, as expected, general self-efficacy was positively related to entrepreneurial intentions across both subsamples. Our findings empirically support previous studies conducted across student populations, where general self-efficacy significantly predicted entrepreneurial intentions (Boyd and Vozikis, 1994; Wang et al., 2016).

Contrary to our expectations, career adaptability was not related to entrepreneurial intentions in either university students and job seekers. Our findings did not replicate those of Tolentino et al. (2014) who found career adaptability to be positively associated with entrepreneurial intentions among Serbian business students. One explanation to these findings could be the fact that career adaptability is a composite of four distinct adapt-abilities (concern, control, curiosity, and confidence) that may not contribute in the same manner to entrepreneurial intentions. Moreover, it has previously been demonstrated that although a majority of job seekers show a motivation for the entrepreneurial career, they seem to be discouraged by perceived environmental barriers regarding this option, and would prefer paid employment (Pari, 2014). Indeed, perceived barriers are subject to individual interpretations and may inhibit intentions (e.g., entrepreneurial intentions) and, thus, the ability of individuals to implement such career choices (Lent et al., 1994). In addition, entrepreneurship was found to be deeply intertwined with social, political, and economic variables, and the transition from intentions to a formal business creation is largely dependent on these variables (Boyd and Vozikis, 1994). Unfortunately, Togo does not appear to be stable in these variables, which may explain the fact that individuals may not systematically activate adaptive resources regarding entrepreneurial intentions even if they scored higher on this career outcome. If career adaptability appeared not to have a direct link with entrepreneurial intentions, career adaptability could have an impact on the ability of people to implement these intentions. However, this would need further investigations.

### The Link Between Self-Perceived Employability and Entrepreneurial Intentions

As expected, self-perceived employability was positively related to entrepreneurial intentions in the two subsamples. According to our findings, the extent to which either university students or job seekers perceived themselves as employable affected their intentions of becoming an entrepreneur. Employable individuals have been described as being likely to handle difficult situations and cope with change (Berntson et al., 2008). Moreover, as documented by several researchers, venture creation requires similar abilities (Wilson et al., 2007). This may explain the positive association between both career outcomes. Our findings suggested that higher levels of perceived employability were associated with higher levels of intentionality regarding venture creation. Furthermore, this path was tested within the hypothesized model. Results suggested no significant contribution of personal resources to entrepreneurial intentions via perceived employability.

### Implications for Educational Policies and Career Counseling

Our findings regarding the relationship between personal resources and career outcomes have several implications for educational policies and career counseling in the Togolese context. The strong and significant influence of career adaptability and self-efficacy on perceived employability has been seen for the two groups. However, such influences appeared to less systematically impact entrepreneurial intentions, which may be more context and learning-dependent. This could indicate that public policies should support entrepreneurship by means of structural, macroeconomic, and educational measures. Concerning the impact of educational or training measures, Liñán and Fayolle (2015) cite several researchers who have found that entrepreneurial education has a significant positive effect on entrepreneurial intentions and, later, on venture creation. Moreover, psychology-based entrepreneurship training financed by the World Bank has shown great potential to impact business growth and sustainability for Togolese entrepreneurs (Campos et al., 2017). In fact, the government of Togo has designed and offered entrepreneurship training to young graduates for several years, but few graduates were able to attend these rather exclusive trainings. Moreover, a 7-month entrepreneurship training program is provided at *Maison de l’Entrepreneuriat* [Entrepreneurship House] at the State University of Lomé and costs 16 times the yearly registration fees for a bachelor’s degree. This may prevent some university students from attending, as the lack of financial means is reported as the most salient perceived career barrier in Togo (Atitsogbe et al., 2016). Entrepreneurship training might be more usefully included as a discipline at universities based on a credit-earning system so that students from various disciplines could benefit from these courses. Similar programs could be highly valuable for high school students as well, as only 12.3% will later enroll in higher education (OECD, 2016).

The results of this investigation underscore the important role of career adaptability and self-efficacy on self-perceived employability. Although this latter construct is subjective, it might be largely influenced by the willingness of people to self-manage their careers. For this reason, career interventions designed for university students and job seekers should aim at strengthening their adapt-abilities. Career construction theorists have highlighted the importance of individuals’ recognition of environmental barriers, development of coping strategies through a mobilization of adapt-abilities (i.e., concern, control, curiosity, and confidence) to self-manage their career and produce significant changes in their lives. These are the focal points that career counselors in contexts subject to extreme unemployment and which seem to be hostile to entrepreneurship should develop in their practice (Atitsogbe et al., 2016).

### Limitations and Future Research

Several limitations regarding this study should be mentioned. The lack of a relationship between career adaptability and entrepreneurial intentions could be attributed to the characteristics of the population studied. Regarding this point, first, it is important to keep in mind that the type of education or the education program considerably influences entrepreneurial intentions (Yıldırım et al., 2016). It is likely that students and recent graduates from economic, business or technical majors would be more skilled to implement entrepreneurial behaviors given the content of their academic programs that may include management courses for example. Our findings suggesting no mean differences between participants who enrolled or graduated in such fields and those from other fields should be taken with caution as the sample sizes considered were relatively small. Further research with larger samples and considering the type of education as a moderator may provide insight into this issue. Moreover, it is possible that our samples consisted of a high proportion of university students and job seekers disadvantaged in the support available from family and friends regarding entrepreneurial intentions. In fact, support from family members and friends has been found to be a significant predictor of entrepreneurial intentions (Liñán and Chen, 2009). Other characteristics such as family background or perceived barriers to entrepreneurship could bring insight into this issue. Hence, adapting and testing the whole entrepreneurial intentions model (Liñán and Chen, 2009) for Togolese university students, job seekers or other populations could provide insights into the role of personal and contextual determinants of entrepreneurial intentions in this country. Finally, only job seekers who attended training provided by the National Employment Agency were recruited. Such training may put the job seekers in a job-seeking state of mind to the detriment of entrepreneurial orientations. Further investigations including those recruited in other contexts would strengthen the generalizability of results regarding the predictive role of career adaptability on entrepreneurial intentions in this population. Career adaptability is a composite of multiple adapt-abilities that may not contribute equally to entrepreneurial intentions, which could explain the lack of association between these constructs. Later studies could investigate the contribution of each of the four adapt-abilities to entrepreneurial intentions in the Togolese context. Finally, perceived employability can’t be considered as a mediator between personal resources and entrepreneurial intentions. In fact, perceived employability and entrepreneurial intentions are only weakly linked.

## Conclusion

This study addressed the role of personal resources on career outcomes of perceived employability and entrepreneurial intentions in West African populations and opened avenues for further investigations. Moreover, investigating the relationship between these two career outcomes among university students and recent graduates seeking work, especially in countries with high unemployment might provide insights into the occupational integration challenges in such contexts. The study contributed filling the gap that can be observed in the literature regarding the lack of studies on the contribution of personal resources to career outcomes in Sub-Saharan Africa. Furthermore, it highlighted the usefulness of career adaptability and to a lesser extent self-efficacy as predictors of perceived employability in populations facing extreme unemployment in an economically constrained context. It showed that self-efficacy specifically was linked to entrepreneurial intentions. Finally, the study showed that self-perceived employability covaried positively with entrepreneurial intentions in university students and job seekers in Togo.

## Author Contributions

KA and JR designed the work. NM and PP collected the data from job seekers and university students, respectively. KA analyzed the data supervised by JR, and wrote the manuscript. LS and JR reviewed the manuscript. NM and PP approved the manuscript.

## Conflict of Interest Statement

The authors declare that the research was conducted in the absence of any commercial or financial relationships that could be construed as a potential conflict of interest.

## Appendix 1

The French version of the Self-Perceived Employability Scale.

(1) J’obtiens des notes élevées dans mes études/I achieve high grades in relation to my studies.
(2) Je considère mon travail académique comme une priorité absolue/I regard my academic work as top priority.
(3) Les employeurs sont désireux de recruter les jeunes diplômés de mon université/Employers are eager to employ graduates from my university.
(4) La réputation de mon université est un atout important pour ma recherche d’emploi/The status of this university is a significant asset to me in job seeking.
(5) Les employeurs ciblent les étudiants de mon université pour recruter des personnes dans mon/mes domaine(s)/Employers specifically target this university in order to recruit individuals from my subject area(s).
(6) Mon université a une réputation exceptionnelle dans mon/mes domaine(s) d’étude/My university has an outstanding reputation in my field(s) of study.
(7) Ma filière d’étude attire beaucoup plus de candidats qu’il n’y a de places disponibles/A lot more people apply for my degree than there are places available.
(8) Mon/mes domaine(s) d’étude est/sont associé(s) à un statut social très élevé/My chosen subject(s) rank(s) highly in terms of social status.
(9) Les personnes exerçant l’emploi que je vise sont très demandées sur le marché du travail/People in the career I am aiming for are in high demand in the external labor market.
(10) Mon diplôme est perçu comme permettant d’accéder à des emplois très convoités/My degree is seen as leading to a specific career that is generally perceived as highly desirable.
(11) De façon générale, il y a actuellement une forte demande pour les diplômés/There is generally a strong demand for graduates at the present time.
(12) Il y a beaucoup de postes vacants dans la zone géographique où je souhaiterais travailler/There are plenty of job vacancies in the geographical area where I am looking.
(13) Je peux facilement trouver des opportunités dans le domaine que j’ai choisi/I can easily find out about opportunities in my chosen field.
(14) Les compétences et aptitudes que je possède sont ceux que les employeurs recherchent/The skills and abilities that I possess are what employers are looking for.
(15) Généralement, j’ai confiance de réussir les entretiens d’embauche ou concours/I am generally confident of success in job Interviews and selection events.
(16) Je crois pouvoir obtenir n’importe quel emploi pour autant que mes compétences et expériences semblent suffisamment appropriées/I feel I could get any job so long as my skills and experience are reasonably relevant.
